# Self-harm on a specialist adult eating disorder unit: a retrospective cohort study of patient characteristics and outcomes

**DOI:** 10.1192/bjo.2021.687

**Published:** 2021-06-18

**Authors:** Leah Holm-Mercer, Douglas Kohler, Agnes Ayton

**Affiliations:** Oxford Health NHS Foundation Trust

## Abstract

**Aims:**

Deliberate self-harm (DSH) is common but rarely studied among inpatients with eating disorders. We sought to investigate the frequency of DSH among inpatients in a specialist adult eating disorders unit, and the association of DSH with comorbidities and treatment outcomes. We also investigated changes in these parameters during the pandemic.

**Method:**

We included the records of 70 patients consecutively admitted to Cotswold House in Oxford between April 2018 and November 2020. Data were analysed using Microsoft Excel using descriptive statistics. For comparisons, student T-tests were used for continuous variables and Chi-square tests used for categorical variables.

**Result:**

99% of patients were female; their ages ranged from 17 to 67 years (mean 30.7). 81% had a primary diagnosis of anorexia nervosa, and 67% had a history of DSH prior to admission. There was a total of 100 incidences of DSH, of which 12% required transfer to a general hospital for medical treatment.

Frequency of self-harm decreased with time throughout admission (17% self-harming on admission, vs 7% at discharge, p = 0.043).

Compared to those with no history of DSH, patients who self-harmed during admission were more likely to be detained under the Mental Health Act (45% vs 17.4%, p = 0.003), and to have psychiatric comorbidities (85% vs 35%, p = 0.001). Patients whose self-harm required transfer for general hospital treatment had a lower mean discharge BMI (18.18kg/m2 vs 20.23kg/m2, p = 0.039), longer admission (105.9 days vs 78.1 days, p = 0.037), and gained weight at a slower rate (0.26kg/m2/week vs 0.43kg/m2/week, p = 0.048) than those who did not require transfer.

During the pandemic, the frequency of DSH doubled on the ward. Overall outcomes were similar, however mean length of admission was lower during the pandemic (67.83 vs 89.94 days, p = 0.046), and patients regained weight more rapidly (0.43kg/m2/week vs 0.28kg/m2/week, p = 0.003) than prior to it.

**Conclusion:**

Self-harm during admission was seen in 29% of patients and was associated with the presence of comorbid psychiatric diagnoses. The frequency of DSH much reduced between admission and discharge, suggesting a beneficial effect of treatment. Medical transfer for DSH, considered as a proxy measure for severity, predicted poorer outcomes in weight restoration. We also noted an increase in rates of DSH during the pandemic, which may have resulted from a combination of increased psychosocial stressors and a reduction in admission capacity in eating disorder units.

